# Emerging Approaches to Complement Low-Dose Computerized Tomography for Lung Cancer Screening: A Narrative Review

**DOI:** 10.7759/cureus.27309

**Published:** 2022-07-26

**Authors:** Bradley Maller, Tawee Tanvetyanon

**Affiliations:** 1 Internal Medicine, Virginia Commonwealth University, Richmond, USA; 2 Thoracic Oncology, Moffitt Cancer Center, Tampa, USA

**Keywords:** lung cancer, methylation profile, volatolomics, radiomics, lung cancer screening

## Abstract

Lung cancer screening by low-dose computed tomography (LDCT) can save lives. Nevertheless, the test suffers from low accuracy. Improving its accuracy will reduce unnecessary invasive procedures and allow lung cancer treatment to be delivered sooner. This review describes the principles, advantages, and disadvantages of selected emerging modalities potentially useful to improve the accuracy of LDCT. A literature search was conducted using PubMed and Google scholar for relevant publications. We identified four key emerging approaches: radiomics, breath analysis, urine test, and blood test. Radiomics, which uses a computer program to extract various radiological features from radiographic images, holds the potential to improve the accuracy of LDCT. However, to date, there remains no adequately validated system. Breath analysis and urine tests represent a noninvasive and convenient means of screening by detecting substances such as volatile organic compounds associated with lung cancer. However, the results can be confounded by diets, medications, and concurrent medical conditions. Finally, a blood test to screen for protein biomarkers or methylation profiles such as Galleri® has high specificity. However, its sensitivity is low, especially for detecting early-stage lung cancer. Furthermore, the cost for mass public use can be significant. Based on our review, blood tests may have potential for future clinical utility. Its high specificity may be useful to rule in a suspicious lung nodule as malignant, so that other additional tests can be omitted. Data from a well-designed clinical trial will be needed to understand the clinical utility of this strategy.

## Introduction and background

Lung cancer remains the leading cause of cancer death [1]. Early detection and prompt treatment can reduce mortality from lung cancer [2]. In 2014, the US Preventive Services Task Force began recommending lung cancer screening with low-dose computed tomography (LDCT) among high-risk smokers [3]. Based on findings from the National Lung Screening Trial (NLST), the mortality risk reduction by LDCT screening was approximately 20%. Subsequent large studies have also substantiated the findings [4]. The National Comprehensive Cancer Network guideline currently recommends LDCT for all adults aged 50 years or older with at least a 20-pack-year smoking history [5].

Nonetheless, there are several limitations with LDCT. One of the key issues is the high rate of false positivity. At baseline screening, the false positive rate is approximately 20-25% [3,4]. False-positive results can increase the utilization of unnecessary procedures or tests. At the least, it can create anxiety among participants. False positivity stems from the fact that benign pulmonary nodules cannot be readily distinguished visually from early-stage lung cancer [6]. Currently, the American College of Radiology uses the Lung Imaging Reporting and Data System (Lung-RADS) to classify lung nodules based on their size and growth rate [7]. False-positive rate decreases when the size cut-off for the definition of a suspicious nodule is increased [8]. In addition, previous CT scan images can be useful. Patients who had previous chest CT scans, such as those with a history of other treated malignancies, have decreased positive findings on LDCT because the stability of nodule size or growth rate can be elucidated over time [9]. However, waiting to observe a pulmonary nodule over time can be harmful if the nodule is, in fact, malignant. There is a need to improve the accuracy of LDCT.

In this article, we reviewed the literature on emerging approaches to refine the accuracy of LDCT. For each approach, we describe the principle and summarize available data relevant to lung cancer screening. We finally discuss the future potential of these approaches.

## Review

Radiomic features

Some unique radiographic features can be used to differentiate benign from malignant nodules. For example, spiculation or irregularity is known to be associated with malignant nodules. To utilize this feature, one will need to be able to reliably quantify the degree of irregularity. Radiomics emerges as a potential solution. Radiomics is the science of describing various radiological features in quantitative variables via the help of computer programs [10].

The principle of radiomics lies in the extraction of radiomic features. Some features cannot be easily understood and merely reflect a subtle interplay of several other features. In general, there are three types of features. The first-order features describe the distribution of all the voxel (i.e. 3-dimensional pixel) values in the CT images. These are histogram-based properties detailing the mean, median, maximum, and minimum values of the voxel intensities on the images. The second-order features are textural features, which are obtained by calculating the spatial relationship between voxels. Finally, the higher-order features are derived by the mathematical transformation of the images. There are various methods to derive the higher-order features contributing to the growing number of publications in this field [11].

As an example, in a study of 72 patients: 40 with lung cancer and 32 with benign lung nodules, images from CT scans were contoured by the investigators and submitted to automated software, resulting in 750 radiomic features. Of these, the investigators found that four features can be used to differentiate lung cancer from benign nodules with an accuracy of 84% [12]. More recent studies have integrated radiomics features with deep learning technology. These include convolutional neural networks [13], artificial neural networks [14], and computer-aided detection (CAD) [7]. Some investigators have reported the sensitivity for lung cancer detection as high as 96% with CAD, compared to 68% as read by a single radiologist [7].

While the science of radiomics is progressing, there are several issues that draw criticism. For example, the lack of clinical or biological correlates with some of the radiomics features raises the possibility that the features may be obtained by chance and will not be useful in other datasets [15]. In fact, some radiomics models appear highly sensitive to variations in image acquisition protocols, making it difficult to replicate. Additionally, radiomics approaches have been developed by several investigator groups almost simultaneously and to date, there has not been a single, well-validated, and widely adopted system. Furthermore, many radiomics models were developed from a small set of images obtained from patients who may not necessarily represent the same population who undergoes LDCT screening [12].

Breath analysis

The concept of volatolomics, an analysis of body fluid for volatile organic compounds (VOC) that emanate from cancer cells or their microenvironment, has been recognized since ancient times. Physicians in the Roman empire used their sense of smell to diagnose disease. Examples include uncontrolled diabetes associated with an acetone odor, liver failure linked with a fish-like smell, and renal failure identified by a urine smell [16]. In oncology, the amount of VOC released from cancer is very small. However, some investigators have described the use of trained dogs to detect malignancies including melanoma, bladder cancer, and lung cancer [17]. A combination of VOCs performs better than a single VOC [17]. While the type and concentration of VOCs can vary from person to person, the close similarity between VOC profiles in each disease gives rise to the concept of discriminative volatolomic signatures [16]. For example, one study has reported a volatolomic signature in lung cancer to include styrene, decane, isoprene, benzene, undecane, 1-hexene, hexanol, propyl benzene, 1,2,4-trimethyl benzene, and heptanal, methyl cyclopentane [17].

Several methods of VOC analysis have been studied including gas chromatography-mass spectrometry, ion mobility mass spectrometry, quartz microbalance, solid phase microextraction, colorimetry, and gold particle nano-sensor [17]. These methods exploit the differential characteristics of VOCs to separate them. For the detection of VOC emanating from lung cancer, the most studied analyzing method has been with a gold nanoparticle. While promising, these techniques demonstrate variable sensitivity and specificity, influenced by both clinical and environmental factors including age, smoking history, and the method of sample collection [17]. Furthermore, cancer cells have been found to have variable VOC patterns, and individual VOCs can be associated with multiple diseases. Decane, for example, is a VOC that has been found in lung cancer as well as liver cancer [16]. Nonetheless, a combination of VOCs or VOC profiles can improve accuracy [17]. Newer approaches include the integration of machine learning. In a small validating study using machine learning of VOC profile obtained from a conductive polymer sensor to diagnose lung cancer, the prediction models showed overall accuracies greater than 90% [18].

Volatolomics still faces major challenges, and several steps will need to be successfully implemented before this technology can come to clinical arenas. First, the timing and method of breath sampling need to be standardized [16,17]. Second, an algorithm needs to be implemented to overcome confounding variables such as age, diet, genetics, and cigarette smoking [18]. Third, once the reliability of a VOC profile is established, a larger validation study, both internal and external will need to be performed. Although volatolomics is promising, this approach still requires much further development [16].

Urine test

A number of substances or biological activities associated with lung cancer can be traced from urine. For example, VOCs or the DNA fragment released from lung cancer can be detected [19]. Furthermore, an abnormally upregulated protease activity associated with some lung cancers can be measured in urine. By first administering an activity-based nano-sensor to a person, the protease activity can be recorded when the sensor is retrieved later from the urine [20,21].

Several studies have reported the utility of urine VOC in detecting lung cancer. In a recent systematic review and meta-analysis, the authors identified 13 studies including 1266 participants testing VOC profiles in five different cancers [21]. The authors found a major inconsistency in the results owing to the heterogenicity of the study design and methodology. For example, a study comparing the concentration of urinary VOCs in patients with lung cancer versus controls demonstrated that VOC profiles are different, not only between lung cancer and benign nodule but also among different histological subtypes of lung cancer [20]. One unique study used trained canines to detect VOC markers in urine samples from lung cancer patients, healthy controls, and patients with non-malignant pulmonary conditions. The results showed a sensitivity from 45% to 73% and a specificity of 89% to 91% for lung cancer diagnosis [22]. In addition to VOC, abnormal DNA methylation due to lung cancer can be recovered from urine. Liu et al. analyzed DNA methylation as a means to screen for NSCLC. The results showed that independent of age, race, and smoking pack-years, the presence of CDO1, TAC1, HOXA9, and SOX17 in urine was significantly associated with NSCLC. When at least three genes were methylated in urine analysis, lung cancer was diagnosed with a sensitivity of 93% and a specificity of 30% [23].

Arguably the most novel technology in urine testing is the use of nano-sensor. Protease activity is commonly dysregulated in cancer [24]. In lung cancer, an abnormal protease activity can be detected among adenocarcinoma with KRAS or TP53 mutation, common genetic alterations in lung cancer [25]. As such, rather than relying on the detection of endogenous biomarkers released into the urine, nano-sensors can be administered to amplify and detect the activity of aberrant protease and the sensors can be recovered from urine, known as urinary reporters [19]. In a pre-clinical study, an assay was coated with protease peptide substrates conjugated to mass-spectrometry-encoded reporters. The nanoparticles accumulate in tumors where the tumor-associated proteases cleave the substrates, thereby releasing the reporters. The reporters subsequently diffuse into the bloodstream and are excreted in the urine. Comparing the mouse models to control mice, this approach was able to detect lung adenocarcinoma with 100% specificity and 81% sensitivity. Though promising, this approach will need further clinical development [19].

Urine detection of lung cancer has its own unique strengths and weaknesses. Similar to breath volatolomics, urine testing can be confounded by medical illnesses [20]. Nevertheless, the DNA collected in urine samples is stable for longer compared to other bodily fluids and large sample volumes can easily be collected to increase the sensitivity [23].

Blood test

Blood tests can be a useful approach to refine the accuracy of LDCT screening. Most of the early studies involving blood-based biomarkers focused on detecting specific mutations associated with lung cancer via circulating tumor DNA (ctDNA) released from lung cancer cells. More recently, studies are investigating the use of methylation profiles in the circulating cell-free DNA (cfDNA). Although cfDNA is not necessarily ctDNA, the methylation profile in ctDNA can be useful to predict the existence of lung cancer. Abnormal DNA methylation may develop early in the course of tumorigenesis [23]. Methylation analysis of cfDNA is quickly emerging as an attractive approach given its robust data output and cost efficiency [26].

In a prospective case-control study by Liu et al., a targeted methylation analysis of cf DNA obtained from over 100,000 informative methylation regions among 6689 participants was performed to potentially detect over 50 cancer types, including lung cancer. For overall cancer detection, the developed platform was reported to have increased sensitivity with increasing stages of cancer [26]. The platform, known as multi-cancer early detection (MCED) tests utilized a machine learning technology. In a validation study consisting of 4077 participants, 2823 with cancer and 1254 without cancer, the MCED test had a sensitivity of 51.5% and specificity of 99.5% across all cancer types [27]. When focusing on a subset of lung cancer, the sensitivity of MCED was 74.8% for all lung cancer stages. However, its sensitivity was only 21.9% for stage I lung cancer. Currently, the MCED test is commercially available known as Galleri® and a randomized clinical trial is being conducted in the United Kingdom; however, the test has not been reviewed by the United States Food and Drug Administration [27].

Protein blood-based biomarkers such as autoantibody have been proposed as a way to diagnose lung cancer in an early stage [28]. Blood-based biomarkers are also being studied to assess their effectiveness in risk-stratifying pulmonary nodules detected on LDCT. For example, the combination panel called 4MP, which includes proteins pro-SFTPB, CA125, CYFRA 21-1, and CEA, showed a good predictive value for cancer. Other biomarkers such as 4MP when used along with LDCT screening may help reduce the number of false-positive screens [29].

Blood-based analyses do have a clear limitation in lung cancer screening; i.e. low sensitivity. It has been estimated that to achieve high-sensitivity detection of ctDNA in stage I-II cancer patients, a large (>80 mL) volume of blood will be needed with current methodologies [30]. In general, the heterogeneity of lung cancer deters the establishment of a single blood-based test to detect early-stage lung cancer [28]. However, given the promise of targeted DNA methylation analysis, large-scale studies will be needed to further elucidate the clinical utility as well as the economic impact of targeted methylation analysis of cfDNA.

Discussion

We have presented four distinct methodological approaches to help improve the accuracy of LDCT screening (Table 1). Radiomics has the potential to enhance LDCT screening by defining various radiological features. Breath analysis, by characterizing VOC profiles, is being studied for its utility as a non-invasive test that can distinguish between lung cancer and benign nodule. Urine testing, similar to breath analysis, employs a non-invasive test strategy to analyze VOC profiles. Urine testing is also being studied to detect abnormal protease activity in lung cancer. Finally, blood testing has also shown promise by detecting the blood-based biomarkers, such as protein biomarkers, ctDNA, and hypermethylation detection via cfDNA associated with lung cancer.

**Table 1:** Summary of approaches

Approaches	Principle	Key strengths	Key weaknesses
Radiomic feature	Lung cancer has morphologically unique features which can be described using radiomic features	Utilizing images obtained from LDCT scan	Lack of widely accepted system to extract radiomic features and validation studies
Breath analysis	Lung cancer produces volatile compounds that can be detected from exhaled breaths	Most convenient way to obtain specimens	Breath samples can be contaminated during collection and confounded by comorbid illnesses
Urine test	Lung cancer produces substances that can be recovered in the urine	Convenient way to obtain specimens	Urine samples can be confounded by diet, medications, and comorbid illnesses
Blood test	Lung cancer releases unique protein or DNA/RNA into the blood	High specificity	Low sensitivity

Although promising, these modalities do possess notable weaknesses. For radiomics, there is a lack of a well-validated, widely accepted system to extract characteristics from lung nodules. In the breath analysis, the process of collecting, storing, and analyzing a breath sample can be cumbersome and has not been standardized. The main drawback of urine testing, like breath analysis, is that VOC profiles can be confounded by diet, medications, and medical comorbidities, thereby limiting sensitivity and specificity. The primary limitation of blood testing is the ability to detect a very small quantity of biomarkers, especially when the cancer is in a very early stage.

A combination of these modalities has the potential to maximize benefits and minimize limitations. Blood-based molecular biomarkers and radiomics have been employed in combination to diagnose early-stage lung cancer with improved sensitivity and specificity [6]. Similarly, combining information from LDCT and volatolomics can improve the accuracy of lung cancer detection [31]. Further studies will be needed to better understand the most efficacious approach to integrating these modalities into LDCT. While our review aims to provide emerging approaches to complement LDCT screening, it is not meant to provide an exhaustive list. Another promising modality, for instance, is the utility of bronchial washing fluid. Transcriptional profiling of bronchial brushings may enhance the diagnostic sensitivity of bronchoscopy alone, even for peripheral and early-stage pulmonary lesions [32,33].

## Conclusions

In summary, many emerging approaches can be useful to complement LDCT screening, including radiomics, urine testing, breath analysis, and blood testing. With future advancements in technology and the continued development of artificial intelligence, LDCT can be augmented by these new strategies to save even more lives.
